# Mechanics rules cell biology

**DOI:** 10.1186/1758-2555-2-16

**Published:** 2010-07-08

**Authors:** James HC Wang, Bin Li

**Affiliations:** 1 MechanoBiology Laboratory, Department of Orthopaedic Surgery, University of Pittsburgh School of Medicine, 210 Lothrop St, BST E1640, Pittsburgh, PA 15213, USA; 2 Orthopedic Institute, Soochow University, 708 Renmin Rd, Suzhou, Jiangsu 215007, China; 3 Department of Orthopedics, The First Affiliated Hospital of Soochow University, 188 Shizi St, Suzhou, Jiangsu 215006, China

## Abstract

Cells in the musculoskeletal system are subjected to various mechanical forces *in vivo*. Years of research have shown that these mechanical forces, including tension and compression, greatly influence various cellular functions such as gene expression, cell proliferation and differentiation, and secretion of matrix proteins. Cells also use mechanotransduction mechanisms to convert mechanical signals into a cascade of cellular and molecular events. This mini-review provides an overview of cell mechanobiology to highlight the notion that mechanics, mainly in the form of mechanical forces, dictates cell behaviors in terms of both cellular mechanobiological responses and mechanotransduction.

## 1. Introduction

Mechanical forces act on humans at different levels, from the body as a whole to individual organs, tissues, and cells. It is well known that appropriate mechanical loads are beneficial to bone and muscle by enhancing their mass and strength. On the other hand, excessive mechanical forces can also be detrimental; for example, excessive mechanical loading of tendons plays a major role in the development of tendinopathy [1,2]. Thus, mechanical forces have a profound effect on tissue homeostasis and pathophysiology. The central players in the human body's response to mechanical forces are various types of mechano-sensitive cells. Examples of such cells include tenocytes in tendons, fibroblasts in ligaments and skin, osteocytes in bone, chondrocytes in articular cartilage, and endothelial cells in blood vessels. Mechanical forces induce a wide range of cellular events, including proliferation, differentiation, and gene and protein expression by both adult differentiated and stem cells [3]. This mini-review provides a concise overview of cellular mechanobiological responses, with a focus on cells from musculoskeletal tissues. In addition, mechanotransduction mechanisms, by which cells "convert" mechanical forces into cellular biochemical events, are also briefly reviewed to emphasize the notion that mechanics, mainly in the form of external and internal mechanical forces, plays a vital role in cell biology. Note that readers who are interested in a more broad and in-depth understanding of the role of mechanics in cell biology should consult relevant papers, which are abundant in the literature.

## 2. External Mechanical Forces

External mechanical forces are defined as forces, such as tensile, compressive, or shear stresses, that are applied to cells from their environment. Depending on the cell type, the forces can come in one form or a combination of them. For example, fibroblasts in tendons and ligaments are mainly under tensile stress *in vivo*, while chondrocytes and osteocytes are subjected to compression and shear stress due to fluid flow in addition to tensile forces. In blood vessels, endothelial cells lining the vessel surface are subjected to a combination of tensile stress due to vessel expansion, hydrostatic pressure, and fluid shear stress.

Because of the ability to control experimental conditions, *in vitro *model systems have been developed to investigate cellular mechanobiological responses. In many of these systems, tensile forces are applied to the substrate and hence cause substrate deformation, which in turn loads cells that adhere to the underlying substrate. There are two ways to apply tensile mechanical forces to cells: the substrate may be stretched uniaxially or biaxially. Uniaxial stretching is appropriate for application of mechanical forces to cells originating from tendons (e.g., patellar and Achilles tendons) and ligaments (e.g., anterior cruciate ligament and medial collateral ligament), as these cells are aligned with their long axis parallel to the tendon or ligament and are therefore subjected primarily to uniaxial stretching *in vivo *[4-6]. On the other hand, biaxial stretching is applied to cells that are subjected to tensile forces in all directions *in vivo*, e.g., dermal fibroblasts. A few biaxial stretching systems have been devised, which typically use circular elastic membranes to produce isotropic strains independent of stretching direction [7-10].

Besides tensile forces, compressive forces can also be applied to cells that are subjected to compression *in vivo*. One way of applying compressive forces is through application of hydrostatic pressure [11,12]. Another technique uses direct platen abutment to apply compressive forces to cells. This type of loading system includes unconfined compression, in which constant or low-cycle intermittent loads are delivered by manually applying weights [13,14], and confined compression, in which cells are placed between two platens that are contained by a confining chamber [15-17]. These compressive loading systems can be used to investigate mechanobiological responses of cells in tissues primarily subjected to compression *in vivo*, such as articular cartilage.

While 2-D systems such as those above have provided us with much insightful information regarding cellular mechanobiological responses, they are inherently limited in that they cannot model an *in vivo *tissue environment where cells are surrounded by extracellular matrix (ECM). Therefore, 3-D systems have also been developed that enable cells to reside in a more *in vivo*-like environment, which better preserves cell phenotype. A widely used 3-D system in the area of wound healing research is a cell-populated collagen gel (CPCG) [18,19]. Cells such as fibroblasts exert contraction forces on the surrounding collagen gel, thus remodeling it [20,21]. External mechanical forces can also be applied to CPCGs to study cellular mechanobiological responses [22,23]. Similarly, bioartificial tissues (BATs) were developed to embed tendon cells in collagen gels [24]. The phenotype of tendon cells in BATs is better preserved than in 2-D systems where cells are attached to 2-D substrates. This is generally true for other types of cells as well; e.g. chondrocytes in 3-D cultures retain their phenotype whereas they quickly lose it when cultured in 2-D systems such as a plastic dish [25].

## 3. Internal Mechanical Forces

Internal mechanical forces are the forces generated by cells themselves and are usually referred to as intracellular tension [26,27]. In non-muscle cells, intracellular tension is generated by cross-bridging of actomyosin, a process powered by ATP hydrolysis [28,29]. Such tensile forces are then transmitted to the ECM via focal adhesions [30], and the forces acting on ECM are called cell traction forces (CTFs). CTFs play a vital role in cell mechanobiology, as they function to direct ECM assembly [31], control cell shape [32-34], permit cell movement [35-39], and maintain cellular tensional homeostasis [40-42]. CTFs also deform the ECM network and cause stress and strain in the network, which in turn modulate cellular functions such as gene expression and protein secretion [43,44]. Therefore, CTFs are critical in many fundamental biological processes such as embryogenesis, angiogenesis, and wound healing [3].

In general, mechanobiological investigations rely on cell-substrate adhesion to transmit external mechanical forces to cells. This is because external mechanical forces acting on cells can alter the equilibrium state of internal forces, thereby affecting cellular mechanobiological responses [45].

In addition to mechanical forces, other mechanics parameters such as substrate stiffness also have a profound influence on cell behavior. A striking example is that substrate stiffness alone can direct specific differentiation of human mesenchymal stem cells (hMSCs); soft substrates (0.1-1 kPa) mimicking brain tissues are neurogenic, whereas stiffer substrates (8-17 kPa) mimicking muscle are myogenic. Finally, even stiffer substrates (25-40 kPa) resembling osteoid matrix can induce hMSCs to undergo osteogenic differentiation [46,47].

## 4. Cellular Mechanobiological Responses

Depending on the type of cell and loading conditions, application of mechanical forces to cells affects a spectrum of cellular functions, including cell proliferation, differentiation, gene expression and protein synthesis of ECM components, and production of cytokines and growth factors. For instance, in one study, human tendon fibroblasts were shown to increase their proliferation as well as gene expression and protein production of type I collagen in a stretch magnitude-dependent manner [48]. In another study, when repetitive stretching at a magnitude of 5% and a frequency of 1 Hz was applied to human tendon fibroblasts for one day, cell proliferation increased significantly. When the same conditions were applied for two days, however, cell proliferation was inhibited [49], indicating that stretching-induced proliferation of tendon fibroblasts also depends on stretching duration. Finally, in human periodontal ligament fibroblasts, a 10% cyclic equi-biaxial compression decreased type I collagen mRNA expression and reduced synthesis of fibronectin as well as the amount of total protein; however, the same level of cyclic stretching increased type I collagen mRNA levels and total protein levels [50]. These findings show that tensile and compressive forces with the same magnitude induce differential cellular mechanobiological responses.

In addition to cell proliferation and protein expression, mechanical forces can also induce the expression and production of inflammatory mediators, including COX-2, PGE_2_, and LTB_4_, in a stretching magnitude-dependent fashion [6,51]. In the presence of IL-1β, a potent inflammatory mediator present in injured tissues, 4% cyclic uniaxial stretching decreased COX-2 and MMP-1 gene expression and PGE_2 _production whose levels had been elevated by IL-1β treatment; in contrast, cells under 8% stretching further increased the expression levels of these genes and PGE_2 _production in addition to the effects of IL-1β stimulation [52]. The findings of this study indicate that mechanical loading regulates cellular inflammatory responses in a loading magnitude-dependent manner. These findings suggest that when tissues such as tendons are injured, appropriate levels of exercise could be beneficial as it may reduce the inflammatory response. On the other hand, excessive loading of injured tendons, which may worsen tissue inflammation, could be detrimental. In chondrocytes, mechanical loading has also been found to regulate cellular inflammatory response via the NF-κB signaling pathway [53].

While numerous studies have focused on studying mechanobiological responses of adult cells, efforts have been placed in recent years on investigating mechanobiological responses of stem cells. Accumulating evidence has shown that mechanical forces regulate proliferation and differentiation of stem cells. For instance, various mechanical loads applied to bovine bone marrow stem cells (BMSCs) induce differentiation of the stem cells into different cell lineages, including ligament cells, chondrocytes, myocardial and vascular cells [54-56]. In addition, small-magnitude stretching promotes osteogenic differentiation of hMSCs, whereas large-magnitude stretching induces tenogenic differentiation, as evidenced by up-regulation of genes specific for osteogenesis and tenogenesis, respectively [57]. Moreover, recent studies have demonstrated that cyclic uniaxial stretching not only enhances proliferation of rabbit tendon stem cells (TSCs), but also induces TSC differentiation into tenocyte and non-tenocyte lineages in a loading magnitude-dependent manner [58].

Corresponding to *in vitro *findings, mechanical forces *in vivo*, usually in the form of exercise, also induce various effects on tissues. For instance, exercise increases procollagen expression, collagen synthesis, and interstitial TGF-β concentration in humans [59]. In mice, moderate treadmill running induces the presence of myofibroblasts in tendons, suggesting that active remodeling takes place in response to applied loading on the tendon [60]. On the other hand, excessive mechanical forces acting on tendons *in vivo *cause degenerative changes in tendons (tendinopathy) [1,2].

Since mechanical loads are essential for the development, function, and repair of body components, mechanical conditioning is used in tissue engineering for proper development and functioning of tissue replacement constructs, especially for those bearing mechanical loads *in vivo *[61,62]. These constructs usually consist of deformable 3-D matrices seeded with cells and can be mechanically loaded using specially designed setups. For example, application of uniaxial stretching to 3-D collagen matrices populated with tendon fibroblasts resulted in expression of type I collagen and fibronectin similar to that of native tendons. This indicates that tendon cells residing in a mechanically loaded 3-D construct could be assuming a similar phenotype as those cells in native tendons. Moreover, these constructs were mechanically stronger than their unloaded counterparts [24]. When autogenous tissue engineered constructs of the patellar tendon made of type I collagen sponges and rabbit MSCs were mechanically stimulated, the stiffness of the cell-collagen constructs increased by 1.5 times compared to unloaded constructs [63]. Also, in smooth muscle cell (SMC)-seeded scaffolds, cyclic mechanical stretching for 5-20 weeks stimulated gene expression of SMC elastin and type I collagen. Tensile strength and Young's moduli of constructs were increased by cyclic stretching for 20 weeks, whereas both decreased over time without mechanical stretching [64].

In addition to external mechanical forces, internal mechanical forces can also regulate cell biology in terms of anabolic or catabolic states. For example, reducing internal mechanical forces (or intracellular tension) by releasing collagen gels from attachment to an underlying substrate or using cytochalasin D, a drug that disrupts actin cytoskeleton, changes rat tendon cells from an anabolic to a catabolic state, as measured by the gene expression levels of type I collagen and interstitial collagenase, respectively [65]. Moreover, cells can also use their internal contractile forces to regulate their own proliferation and differentiation [66,67].

Theoretical modeling predicted that accumulation of mechanical stress happens in a layer of cells that adhere to each other, and cells may use the mechanical stress as a feedback signal for their division [67,68]. Using a micropatterned cell aggregate model, concentrated internal mechanical stresses around the perimeter of a cell aggregate were shown to cause endothelial or epithelial cells to proliferate at the perimeter but not in its inner region, where lower mechanical stresses were present [69]. Furthermore, collective internal mechanical stresses were also found to regulate differentiation of a cell aggregate, resulting in spatial patterning of differentiated cells [70,71]. Finally, decreasing intracellular tension or internal mechanical force by limiting cell spreading area has been shown to result in apoptosis [72].

## 5. Cellular Mechanotransduction

**Figure 1:**
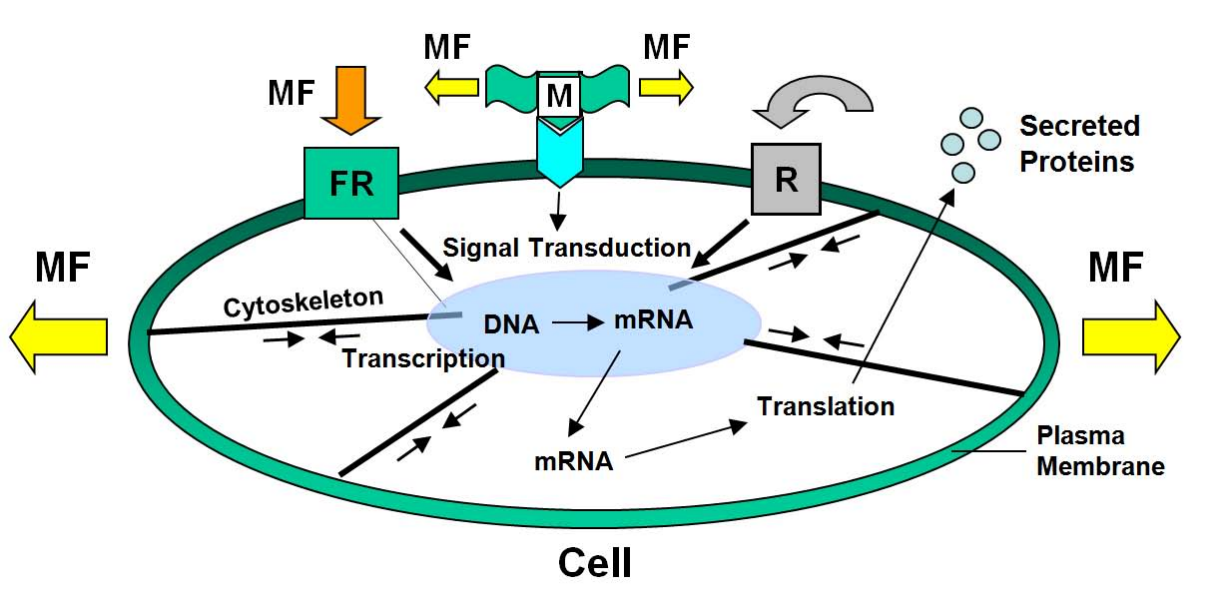
**Schematic illustration of the "mechanical nature" of cellular mechanotransduction mechanisms**. Mechanical forces (**MF**) can induce mechanotransduction by directly altering conformation of an extracellular matrix (**ECM**) protein and integrin configuration and transmitting forces to the cytoskeleton and nucleus, thus eventually affecting transcription and translation. Also, mechanical forces can unfold a domain of the extracellular protein (**M**) and expose a cryptic site that may serve as an activating ligand for a cell surface receptor, resulting in a series of signaling events. Also, when mechanical forces are applied to "force receptors" (**FR**), such as integrins and G proteins, they initiate signal transduction, resulting in transcription followed by translation. As a result, soluble factors are secreted into the ECM, which act on the receptor (**R**) and then initiate a cascade of signaling events. Note that double arrows indicate intracellular tensions in the actin filaments. (Modified with permission from Wang and Thampatty, **Fig. four **in *Encyclopedia of Biomaterials and Biomedical Engineering*, 2008, p.1783-1793, Taylor & Francis).

In order for a cell to respond to mechanical forces, the mechanical forces must be converted into chemical signals inside the cell to elicit a cascade of cellular and molecular events. Such a process is termed cellular mechanotransduction (Fig. 1).

While the mechanisms of cellular mechanotransduction are still not completely understood, it is generally accepted that external mechanical forces acting on ECM have to be transmitted into a cell through integrin-mediated adhesions [73,74]. Integrins, which contain both a large ECM domain responsible for binding substrates and a cytoplasmic domain, are the main adhesive receptors and mechanotransducers that link the cytoskeleton to the ECM [26,75]. Therefore, the ECM-integrin-cytoskeleton pathway plays a major role in the mechano-signaling process. In a "tensegrity" model, mechanical forces applied to the cell membrane are directly and immediately transmitted to the nucleus through the inter-connected cytoskeleton composed of actin filaments, microtubules, and intermediate filaments [76]. Such a model is supported by the finding that application of mechanical stress to integrins altered the cytoskeleton and activated gene expression in a stress-dependent manner [77-79]. Using a FRET-based cytosolic Src reporter in a living cell, local stress was shown to induce rapid activation (< 0.3 sec) of Src at remote cytoplasmic sites; thus, a pre-stressed cytoskeleton can rapidly transduce mechanical signals [80].

In addition to integrins and the cytoskeleton, G proteins also function as mechanotransduction molecules [81,82]. Another important component of cellular mechanotransduction is intracellular Ca^2+ ^[83]. Mechanical stretching of fibroblasts and many other types of cells increases the levels of intracellular Ca^2+^, which serves as a secondary messenger [84,85]. In addition, cellular mechanotransduction also involves stretch-activated ion channels (SACs) [86,87]. In response to applied mechanical stresses, SACs open to allow ions like Ca^2+^, Na^+^, and K^+ ^to pass through, thus transducing mechanical signals into activation of intracellular signaling molecules [88]. Finally, recent studies have shown that primary cilia also play an important role in cellular mechanotransduction. In bone cells, for example, primary cilia translate fluid flow into cellular responses independent of SACs [89].

In addition to the roles of many cellular components such as integrin and cytoskeleton in cellular mechanotransduction, researchers are also beginning to understand the mechanisms of how mechanical forces are initially sensed by the cell. In adherent cells, force transmission is primarily dependent on the attachment of cells to ECM molecules such as collagen or fibronectin [90]. Therefore, ECM proteins may function as "force sensors." Mechanical stresses acting on ECM may unfold a domain of the ECM protein, resulting in exposure of its cryptic site, which may serve as an activating ligand for an adjacent receptor [83]. This potential force-sensing mechanism is supported by the finding that small and large forces unfold the weakest domain and the most stable domain of fibronectin, respectively [91]. Besides the conformation change in an ECM protein due to applied external mechanical forces, the cytoskeletal force, or the internal mechanical force, controls α_5_β_1 _integrin switching between relaxed and tensioned states. Such a switch directly controls the strength of α_5_β_1_-fibronectin bond by engaging the synergy site in fibronectin [92].

## 6. Conclusion

Mechanical forces are ubiquitous and are known to greatly influence physiology and pathophysiology in humans. Mechano-responsive cells are responsible for these mechano-effects, as years of intensive mechanobiology research have shown that external mechanical forces influence a wide spectrum of cellular events, including alterations in cell proliferation, differentiation, gene expression, and protein production. It is also now appreciated that internal mechanical forces generated by cells themselves regulate cell biology in terms of metabolic state, cell proliferation and differentiation, etc. Particularly, CTFs, which are the internal mechanical forces transmitted to ECM, regulate many vital cellular functions such as migration and ECM assembly.

The keys to understanding mechanical force-regulated cell biology are cellular mechanotransduction mechanisms by which cells "convert" mechanical force signals into biochemical signals in cells. The role of ECM proteins, integrins, and cytoskeleton in cellular mechanotransduction is now firmly established. Recent studies also point to predominant role of primary cilia in mechanical signal transduction. They also show that mechanical forces may cause mechanotransduction events by altering conformation of signaling molecules, thus affecting their activity and consequently eliciting a cascade of biochemical events such as gene expression.

The fact that mechanics plays a dominant role in cell biology provides a solid foundation and rationale for use of mechanics to improve human health by designing appropriate equipment/instruments, exercise protocols, and rehabilitation regimens. For instance, in sports medicine, such practices will help improve overall performance while reducing and preventing musculoskeletal injuries in athletes. Also, combined use of "bio-interventions" and "mechanics" will further improve the outcome of clinical treatments of musculoskeletal injuries.

## Competing interests

The authors declare that they have no competing interests.

## Authors' contributions

JW and BL drafted and revised the manuscript together. Both authors read and approved the final manuscript.
